# Hospital Contracts and Prices

**Published:** 1910-04-23

**Authors:** 


### Special Departmental Article.

### HOSPITAL CONTRACTS AND PRICES.

### III.—THE PURCHASE OF DRUGS.

**Contributions and questions on matters affecting this department of an institution are invited.**

In previous issues we dealt with provision contracts and the prices at which certain articles of food could be purchased. The next sub-head in the Uniform System of Accounts is "Surgery and Dispensary," and amongst the numerous drugs and dressings there are huge possibilities of variations in price, and watchful care and keen buying are necessary to keep the expenditure down. This sub-head is one where the members of the honorary and resident medical staff can involve the hospital in a good deal of expense. Fancy prescriptions of an expensive nature will soon run up a heavy drug bill, and a charity owes much to the members of its medical staff who, while prescribing with due regard to the object for which the institution exists—viz. the speedy cure or relief of the sick poor—yet exercise thoughtful discretion.

With regard to the purchase of drugs many methods are adopted in the numerous hospitals in the country. Some have a fixed arrangement with a local chemist to supply everything required at the market rate at the time of requirement. Others prepare a monthly or quarterly list of requirements, and invite tenders from all and sundry; others permit only selected firms to tender for their requirements, while again other institutions enter into contracts for such supplies as may be necessary for a fixed period. There is a good deal to be said for all these methods, but as there is no trade in existence in which confidence in the supplying firm is so essential as in the drug trade, and the detection of an inferior article is in many instances so difficult, nay, practically impossible, we are of opinion that hospitals should permit only manufacturing firms of high standing and known repute to tender for their drug supplies. These are sufficiently numerous to promote a healthy competition, and by a careful selection of the various items in the schedule hospitals are able to get bottom prices and yet be sure that they are getting the best articles. By a shrewd purchase at the right moment manufacturing firms are able to put certain of their goods upon the market at a price slightly below their competitors', and the purchasing authorities of a hospital are wise if they take advantage of this slight variation in price.

There are certain items, such as cod-liver oil, which should not be included in any contract. They should be bought in large quantities when at their cheapest, prices for a year's supply being obtained from wholesale dealers.

In these days of huge numbers of operations a great saving can be effected in the drug bill if methylated ether and chloroform be purchased instead of ether and chloroform made from the pure spirit. Very few of the large London hospitals now use these drugs in the more costly form. Chemically, methylated chloroform and ether are absolutely the same as the pure article, and they can be employed with no more risk to the patient, and it seems to be chiefly from sentimental reasons that certain institutions continue to use the more expensive article. The respective prices of methylated and pure chloroform are 1s. 6d. and 5s. 8½d. per lb., and when the consumption in a large hospital approaches half a ton per annum some idea of the enormous saving effected by purchasing the cheaper article is recognised.

Two other drugs which are used in fairly large quantities in most hospitals are bromide of potassium and iodide of potassium. Two years ago the first of these could be purchased at 6d. per lb.; at the present time the price ranges from 1s. 3d. to 1s. 9d. An increase was foreseen a few months ago, and several hospitals laid in large stocks at 10¾d., which has resulted in a considerable saving. Iodide of potassium is being purchased by hospitals at the present time at prices ranging from 7s. to 9s. per lb. There is little or no difference in the quality of these goods, the variation in price being due in great measure to the method of ordering. The purchase of these and many other drugs in a good market in as large quantities as can be kept without deterioration is to be commended.

There is also a considerable variation in the cost of industrial spirit if not purchased direct from the methylator. Turpentine, too, is an item in which a good deal of money can be spent if the price is not carefully watched. The cost of carbolic acid apparently varies considerably when the prices paid at a number of hospitals are compared. It can be obtained at the present time at 4¾d. per lb., the quality and strength being guaranteed, and hospital authorities who are stocking preparatory to a rise are displaying wise foresight.

Where possible, an institution should make its own galenicals. In many institutions, by a little push, it is possible for the dispenser, assisted by the dispensary porter, to fit this in with his other work, and the saving of cost is all in favour of the hospital, as the persons employed in the preparation of these goods would find their time occupied by routine matters if they were not making galenicals, and therefore the filling of their time by this method is a distinct advantage to the institution.

The following are prices which we have recently seen in a six-months' contract, and they appear fair.

| | |
|---|---|
| Bismuth. carb. ... ... ... | 7s. 5½d. per lb. |
| American turps. ... ... ... | 45s. 6d. per cwt. |
| Turps substitute ... ... ... | 1s. 3d. per gal. |
| Extract of malt and oil ... ... | 24s. 6d. per cwt. |
| Acid. boric. ... ... ... ... | 25s. 6d. per cwt. |
| Ol. ricini ... ... ... ... | 4d. per lb. |
| Glycerine ... ... ... ... | 95s. 6d. per cwt. |
| Quinine ... ... ... ... | 7d. per oz. |
| Mag. sulph. ... ... ... ... | 6s. 3d. per cwt. |
| Phenacetin ... ... ... ... | 2¼d. per oz. |
| Soda bi-carb. ... ... ... ... | 8s. per cwt. |
| Zinc oxide ... ... ... ... | 3½d. per lb. |

